# Identification of ferroptosis related markers by integrated bioinformatics analysis and In vitro model experiments in rheumatoid arthritis

**DOI:** 10.1186/s12920-023-01445-7

**Published:** 2023-01-30

**Authors:** Jinjun Xia, Lulu Zhang, Tao Gu, Qingyang Liu, Qiubo Wang

**Affiliations:** Department of Clinical Laboratory, Wuxi 9Th People’s Hospital Affiliated to Soochow University, No. 999 Liang Xi Road, Binhu District, Wuxi, 214000 Jiangsu China

**Keywords:** Rheumatoid arthritis, Differentially expressed genes, Ferroptosis-related genes, Biomarkers

## Abstract

**Background:**

Rheumatoid arthritis (RA) is an autoimmune disease characterized by destructive and symmetrical joint diseases and synovitis. This research attempted to explore the mechanisms involving ferroptosis in RA, and find the biological markers by integrated analysis.

**Methods:**

Gene expression data (GSE55235 and GSE55457) of synovial tissues from healthy and RA individuals were downloaded. By filtering the differentially expressed genes (DEGs) and intersecting them with the 484 ferroptosis-related genes (FRGs), the overlapping genes were identified. After the enrichment analysis, the machine learning-based approaches were introduced to screen the potential biomarkers, which were further validated in other two datasets (GSE77298 and GSE93272) and cell samples. Besides, we also analyze the infiltrating immune cells in RA and their correlation with the biomarkers.

**Results:**

With the criteria, 635 DEGs in RA were included, and 29 of them overlapped in the reported 484 FRGs. The enrichments of the 29 differentially expressed ferroptosis-related genes indicated that they may involve in the FoxO signaling pathway and inherited metabolic disorder. RRM2, validating by the external datasets and western blot, were identified as the biomarker with the high diagnostic value, whose associated immune cells, such as Neutrophils and Macrophages M1, were also further evaluated.

**Conclusion:**

We preliminary explored the mechanisms between ferroptosis and RA. These results may help us better comprehend the pathophysiological changes of RA in basic research, and provide new evidences for the clinical transformation.

**Supplementary Information:**

The online version contains supplementary material available at 10.1186/s12920-023-01445-7.

## Introduction

Rheumatoid arthritis (RA) is a chronic, systemic autoimmune disease whose main pathological features are synovitis with swelling, cartilage destruction and pannus [1]. RA is associated with progressive disability, systemic complications, early mortality, and increased socioeconomic costs [2], among which systemic complications mainly include cardiovascular disease [3], pulmonary interstitial lesions [4], joint deformities [5], malignancies [6], and depression [7]. Epidemiological findings show that the global incidence of RA is 0.5%—1.0%, and the male to female ratio is approximately 1:4 [8]. RA causes both a decline in physical function, quality of life, and social participation, as well as a significant economic burden on the patient's family and society [9]. Despite the availability of numerous biologics and targeted small molecule drugs, there are still many patients who struggle to achieve clinical remission.

In a study in 2003, Stockwell et al. explored the killing effect of various compounds on tumor cells through large-scale screening experiments, and found a new compound, Erastin, which can cause tumor cells with ras mutation to die in a different way from traditional methods [10]. In 2012, the Eastin induced cell death was finally defined as an iron dependent regulated cell death (RCD), named “ferroptosis” [11]. ferroptosis differs significantly from other types of RCD (e.g., apoptotic, necrotic, and autophagic cell death) at the morphological, biochemical, and genetic levels. It is a unique form of non apoptotic cell death dependent on iron and lipotoxicity, triggered by iron catalyzed lipid peroxidation initiated by non enzymatic (Fenton reaction) and enzymatic mechanisms (lipoxygenases) [12]. Recent studies have shown an association between the RA and ferroptosis [13], implying that ferroptosis is involved in the onset and progression of RA. This points a new direction for our current study.

Glutathione peroxidase (GPX) 4, which is dependent on glutathione (GSH), can degrade lipid peroxides. GPX4 failure can lead to iron ion-mediated accumulation of lipid reactive oxygen species (L-ROS), which ultimately leads to ferroptosis [14]. Recent studies have found that ROS, GPX and iron accumulation are closely related to the occurrence and development of RA, which indicates that RA and ferroptosis-related pathological processes are likely to intersect. ROS can be generated in the normal metabolic process of the human body. At physiological concentration, ROS, as an active molecule of metabolism, plays a physiological regulating function, but under pathological conditions, the production and removal of ROS are out of balance, which will cause damage to the human body. The pathogenesis of oxidative stress in RA is that ROS induces the expression of hypoxia-inducible factors and regulates a variety of pro-angiogenic factors in pathological vascular proliferation in RA, among which vascular endothelial growth factor plays a major role [15]. Therefore, we speculate that ROS may affect the occurrence and development of RA through ferroptosis. GPX of the ferroptosis pathway is also linked to RA. In a number of RA drug studies, an increase in the expression of GPX4 was observed along with a decrease in inflammatory factors, mainly TNF-α [16]. In addition, a higher prevalence of RA was reported in the people using deferiprone, and the authors attributed this phenomenon to iron chelator induced iron deposition or free radical generation during iron exchange in the synovium [17]. The mechanism of ferroptosis has now become a new focus in RA research. Although the signaling pathway centered on GPX4 is basically clear, many potential ferroptosis-related targets remain to be discovered.

Identifying and validating the ferroptosis-related genes in RA is valuable for making the definitive diagnosis and deciding the treatment plan. With the increasing bioinformatics progress, the screening of key genes associated with RA has become possible. In our research, we attempted to explore the role of ferroptosis in RA by an integrated bioinformatics analysis. Differentially expressed genes associated with ferroptosis in RA were identified by downloading, processing, and analytical computation of public data. The enrichment analysis and machine learning algorithms were then performed. Combined with the gene comparison of external datasets and the verification of western blot based on the ferroptosis model in vitro, the target molecules were finally determined. The clinical diagnostic value and associated biological functions of the gene were systematically considered. The whole analysis help us deeply comprehend the ferroptosis-related mechanisms of rheumatoid arthritis, contributing to discover new strategies for the currently intractable osteoarticular diseases.

## Materials and methods

**Fig. 1:**
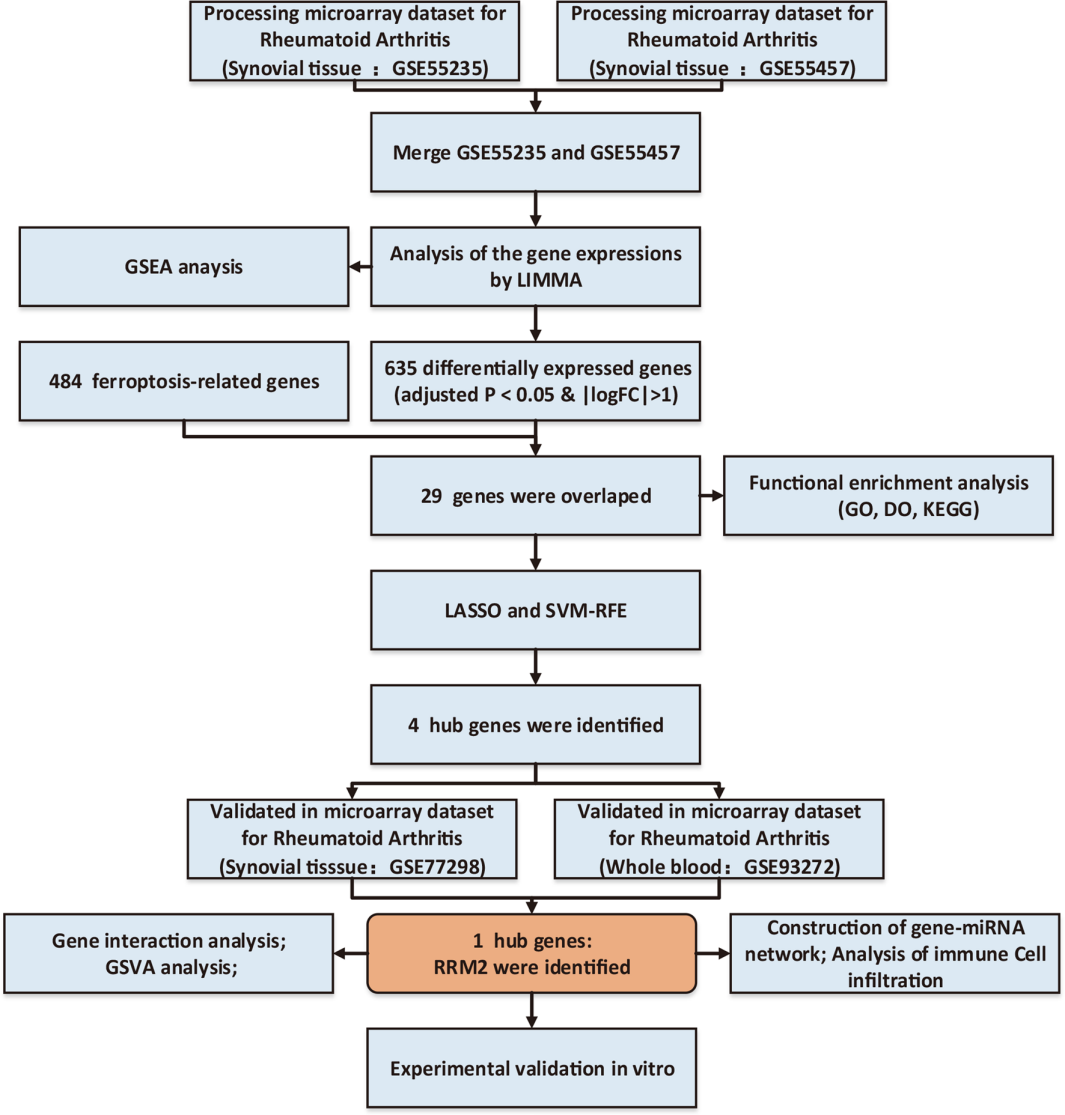
The flowchart for the research

Figure 1 presents the whole flow of this research.

### Microarray data acquisition and processing

A list of 484 ferroptosis-related genes (FRGs), including Driver; Suppressor; and Marker, was obtained from the FerrDb V2 (http://www.zhounan.org/ferrdb/current/) [18]. The specific symbol of these genes are shown in the Additional files 1, 2, and 3.

The GSE55235 dataset [19] (10 synovial tissues from healthy joint; 10 synovial tissues from rheumatoid arthritis joint) and the GSE55457 dataset [19] (10 synovial tissues from healthy joint; 13 synovial tissues from rheumatoid arthritis joint) employed in this work was acquired from the NCBI-GEO database (https://www.ncbi.nlm.nih.gov/geo/). The two gene expression profilings were both based on GPL96 [HG-U133A] Affymetrix Human Genome U133A Array. In addition, the GSE77298 dataset [20] (7 synovial tissues from healthy joint; 16 synovial tissues from rheumatoid arthritis joint) and GSE93272 dataset [21] (43 cases of whole blood from healthy control; 232 cases of whole blood from rheumatoid arthritis patients) were also downloaded, which were both based on the GPL570 [HG-U133_Plus_2] Affymetrix Human Genome U133 Plus 2.0 Array. According these information, probe ID for each gene were transformed into the official gene symbols. When multiple probes corresponded to one gene, the most significant was selected. Some probe ID were discarded if they did not have a corresponding gene symbol. Two expression profile files (GSE55235 and GSE55457) were then merged (after log2 transformation and normalization), and the sva package (version 3.36.0) was used to eliminate the batch effects. The merged dataset (GSE55235 and GSE55457) was used as the training set in our research, while the GSE77298 dataset and GSE93272 dataset were served as the validation set. All the above operations were performed with R software (version 4.0.2).

### Identification of differentially expressed genes (DEGs)

The “limma” package [22] (version 3.44.3) was executed to identify the differentially expressed genes (DEGs) between controls and RA tissues in the merged dataset (GSE55235 and GSE55457). Based on the calculated results, the gene set enrichment analysis (GSEA) enrichment analysis were performed through executing the clusterProfiler package (version 3.16.0) [23]. The “c5.go.v7.4.symbols.gmt” and the “c2.cp.kegg.v7.4.symbols.gmt” were used as the reference gene set. When DEGs met the cutoff criteria: adjusted *P* < 0.05 and |logFC|> 1, they were considered significant. The overlapping targets between the significant DEGs and the 484 FRGs were presented in a Venn diagram, which were defined as differentially expressed genes related to ferroptosis (DFRGs) and used for the further research.

### Enrichment analysis

After getting the above overlapping genes, the Kyoto Encyclopedia of Genes and Genomes (KEGG) pathway, Gene Ontology (GO) [24], and disease ontology (DO) [25] enrichment analysis were subsequently performed through executing the clusterProfiler package (version 3.16.0). The enrich terms with a *P* < 0.05 were considered as statistically significant.

### Screening of the ferroptosis-related biomarkers

To screen the ferroptosis-related biomarkers among the DFRGs, the least absolute shrinkage and selection operator (LASSO) logistic regression and support vector machine-recursive feature elimination (SVM-RFE) were introduced. In brief, the LASSO and the SVM-RFE algorithm were respectively performed with the “glmnet” package (version 4.1–1) and “e1071” package (version 1.7–6) in R software (version 4.0.2). The overlapping genes obtaining from the results of the two algorithms were are preliminarily identified as the ferroptosis-related biomarkers, which were validated by the GSE77298 and GSE93272 dataset. Using the pROC package [26] (version 1.18.0), the receiver operating characteristic (ROC) curve were drew to evaluate its diagnostic value.

### Immune infiltration analysis

The CIBERSORT algorithm [27] was chosen to analyze the differential proportion of 22 types of immune cells in the synovial tissues between the healthy and RA group (GSE55235 and GSE55457; filtered with *P* < 0.05). Further more, the spearman correlation analysis was carried out between the ferroptosis-related biomarkers and the immune cells. The “vioplot” package (version 0.3.5) [28] and the “ggplot2” package (version 3.3.2) [29] was used for the results visualization.

### Gene set variation analysis (GSVA) analysis

To evaluate the potential changes of biological function for the ferroptosis-related biomarkers, we used the GSVA algorithm [30] to comprehensively enrich the related gene set. The “c2.cp.kegg.symbols.gmt” and the “c5.go.symbols.gmt” were used as the reference gene set in our study.

### Construction of gene network

GeneMANIA (http://www.genemania.org) [31] is an online tool for building PPI network. It allows the identification of 20 contiguous proteins of target genes. Herein, GeneMANIA was adopted to construct the gene network of ferroptosis-related biomarkers in RA.

### Prediction of microRNAs (miRNAs)

To search for the potential miRNAs of ferroptosis-related biomarkers, the online databases, including miRanda (http://www.mocrorna.org), miRDB (http://www.mirdb.org/), and TargetScan (http://www.targetscan.org/) [32] were used. The miRNAs identified in all the 3 databases were considered reliable prediction results.

### Cell culture and the establishment of ferroptosis model in vitro

Human chondrocytes (C28/I2 cells) at a density of 1 × 10^4^ cells/well were placed on the 96-well plate, which were maintained in regular DMEM medium (Gibco, United States, Cat No. C11995) supplemented with 10% FBS (Wisent, Canada) in an air saturation of 5% CO2 at 37 °C [33]. Based on this, an in vitro models of ferroptosis was established by Erastin (ferroptosis inducer) with/without blockade of ferroptosis-related biomarkers.

### Cell counting kit-8 (CCK-8) assay

After incubating for 24 h, upper medium was removed, and 20 µL of CCK-8 reagent (Beyotime, Shanghai, China) was added to each well. Under the microplate reader (Bio-Rad, USA), cell viability was evaluated at a wavelength of 450 nm 4 h later [34].

### Protein extraction and western blot validation

C28/I2 cells were lysed in moderate RIPA buffer (Beyotime, Shanghai, China). The supernatants were gathered after incubation (on ice; 30 min) and centrifugation (14,000 g; 4 °C; 5 min). The BCA assay was used for total protein measurement. Proteins were separated in the 10% SDS gel and transfered to the PVDF membranes with two hours blocking (5% non-fat dry milk/TBST solution) [35]. Next, the membranes were sequentially incubated with anti-RRM2 overnight at 4◦C (diluted 1:1000; Cell Signaling Technology, USA) and anti-rabbit secondary antibody for 2 h at indoor temperature (diluted 1:3000; Servicebio, Wuhan, China). ECL kits (Beyotime, Shanghai, China) was used for the visualization of the signal band and data acquisition was done using ImageJ software. The expression of protein was normalized by β-actin.

### Statistical analysis

Statistical analysis were performed with SPSS 23.0 (Chicago, USA). The parametric Student’s t-test was used in the data examination. All tests were two-tailed. It was considered statistically significant when *P* < 0.05.

## Results

### GSEA analysis and genes identification between the DEGs and the FRGs

**Fig. 2:**
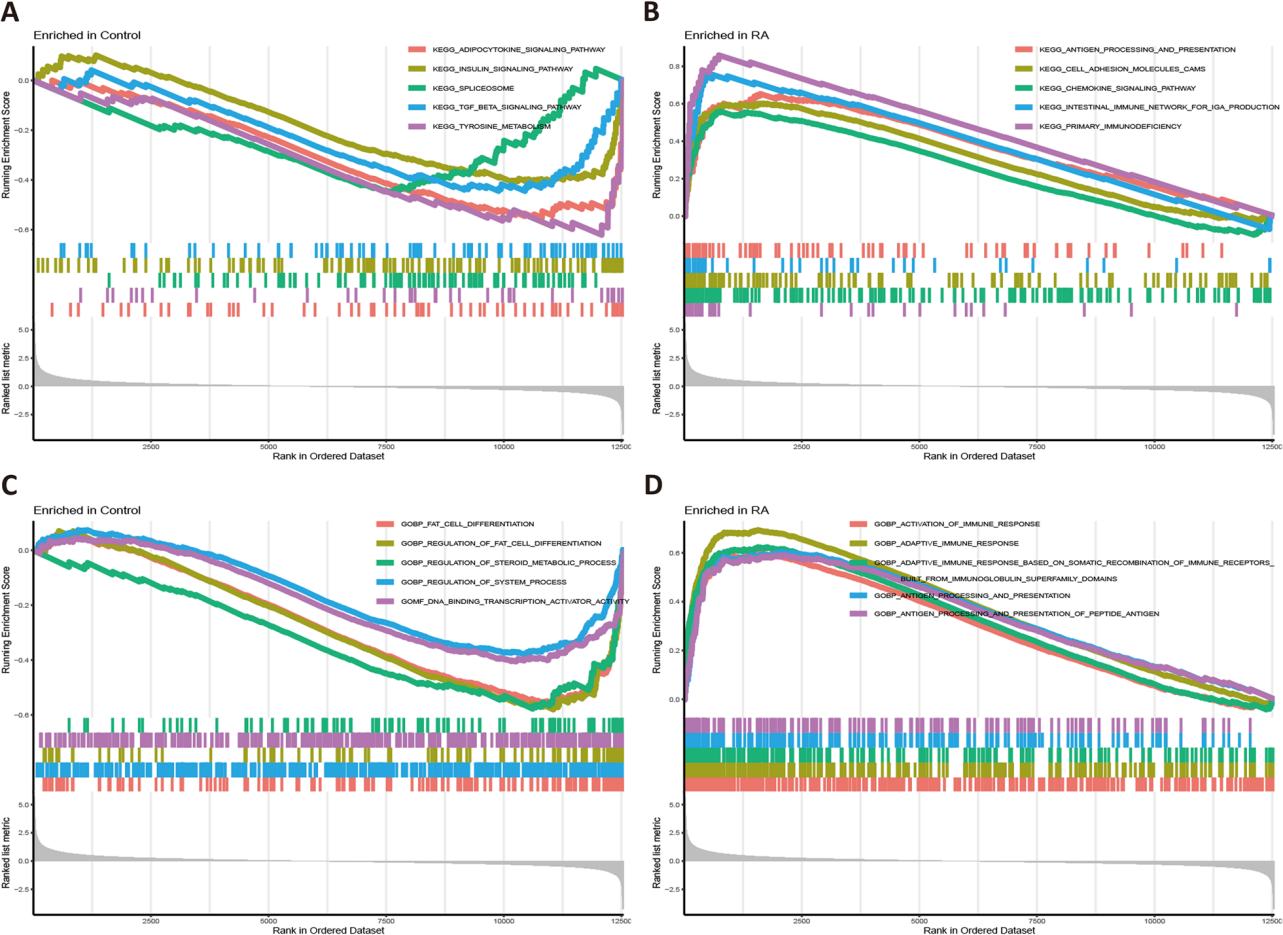
The enrichment result of GSEA. **A** KEGG in the control; **B** KEGG in the RA; **C** BP in the control; **D** BP in the RA

The KEGG enrichments for participated samples in the GSEA conveyed that the controls are more related with ADIPOCYTOKINE SIGNALING PATHWAY, INSULIN SIGNALING PATHWAY, and SPLICEOSOME (Fig. 2A), while the ANTIGEN PROCESSING AND PRESENTATION, CELL ADHESION MOLECULES CAMS, and CHEMOKINE SIGNALING PATHWAY are remarkably in the RA group (Fig. 2B). Likewise, The biological process (BP) enrichments indicated that the FAT CELL DIFFERENTIATION, REGULATION OF FAT CELL DIFFERENTIATION, and REGULATION OF STEROID METABOLIC PROCESS are likely enriched in the controls (Fig. 2C), while the ACTIVATION OF IMMUNE RESPONSE or ADAPTIVE IMMUNE RESPONSE were possibly involved in the RA group (Fig. 2D).

**Fig. 3:**
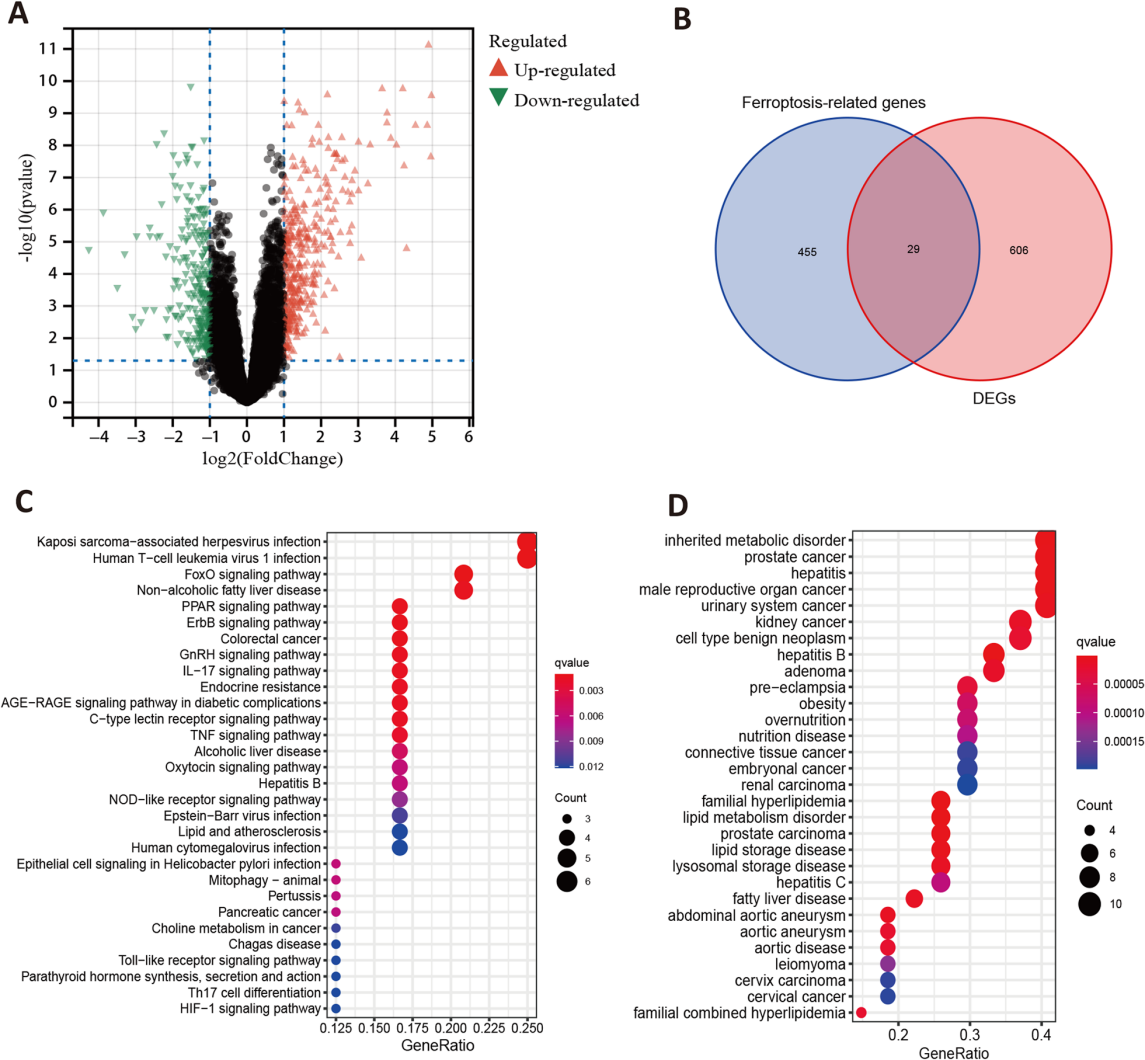
Differentially expressed genes in RA and their relationship with ferroptosis. **A** the volcano map of the DEGs in the RA (adjusted *P* < 0.05 and |logFC|> 1); **B** intersection of the ferroptosis-related genes with the DEGs in RA: a total of 29 overlapping genes; **C** the significant KEGG enrichment terms for the 29 overlapping genes; **D** the significant DO enrichment terms for the 29 overlapping genes

On the other side, a total of 635 DEGs (Additional file 4) were identified between controls and RA synovial tissues (adjusted *P* < 0.05 and |logFC|> 1), including 255 down-regulated and 380 up-regulated genes, which were shown in a volcano map (Fig. 3A). Among the DEGs and the FRGs, there were 29 overlapping items (DFRGs, Fig. 3B). The details of the differentially expressed ferroptosis-related genes were shown in Additional file 5. We used them for the next enrichment analysis.

### KEGG, DO, and GO enrichment analysis for the overlapping genes

As shown in Fig. 3C, 30 significant pathways were displayed. Kaposi sarcoma-associated herpesvirus infection (hsa05167), Human T-cell leukemia virus 1 infection (hsa05166), and FoxO signaling pathway (hsa04068) were highlighted in the KEGG enrichments. Among the 30 significant DO items (Fig. 3D), inherited metabolic disorder (DOID:655) was dominant. On the other side, the GO terms consist of biological process (BP), cellular component (CC), and molecular function (MF). In Additional file 6, we presented their 10 most significant annotations respectively. For example, fat cell differentiation (GO:0,045,444), response to nutrient levels(GO:0,031,667), and regulation of fat cell differentiation (GO:0,045,598) was most remarkable in BP. As for the CC, nuclear membrane (GO:0,031,965), transcription regulator complex (GO:0,005,667), and nuclear envelope (GO:0,005,635) were prominently enriched. In the core enrichments of MF, oxidoreductase activity, acting on single donors with incorporation of molecular oxygen, incorporation of two atoms of oxygen (GO:0,016,702) was the most significant.

### Screening and validation of the hub ferroptosis-related genes

**Fig. 4:**
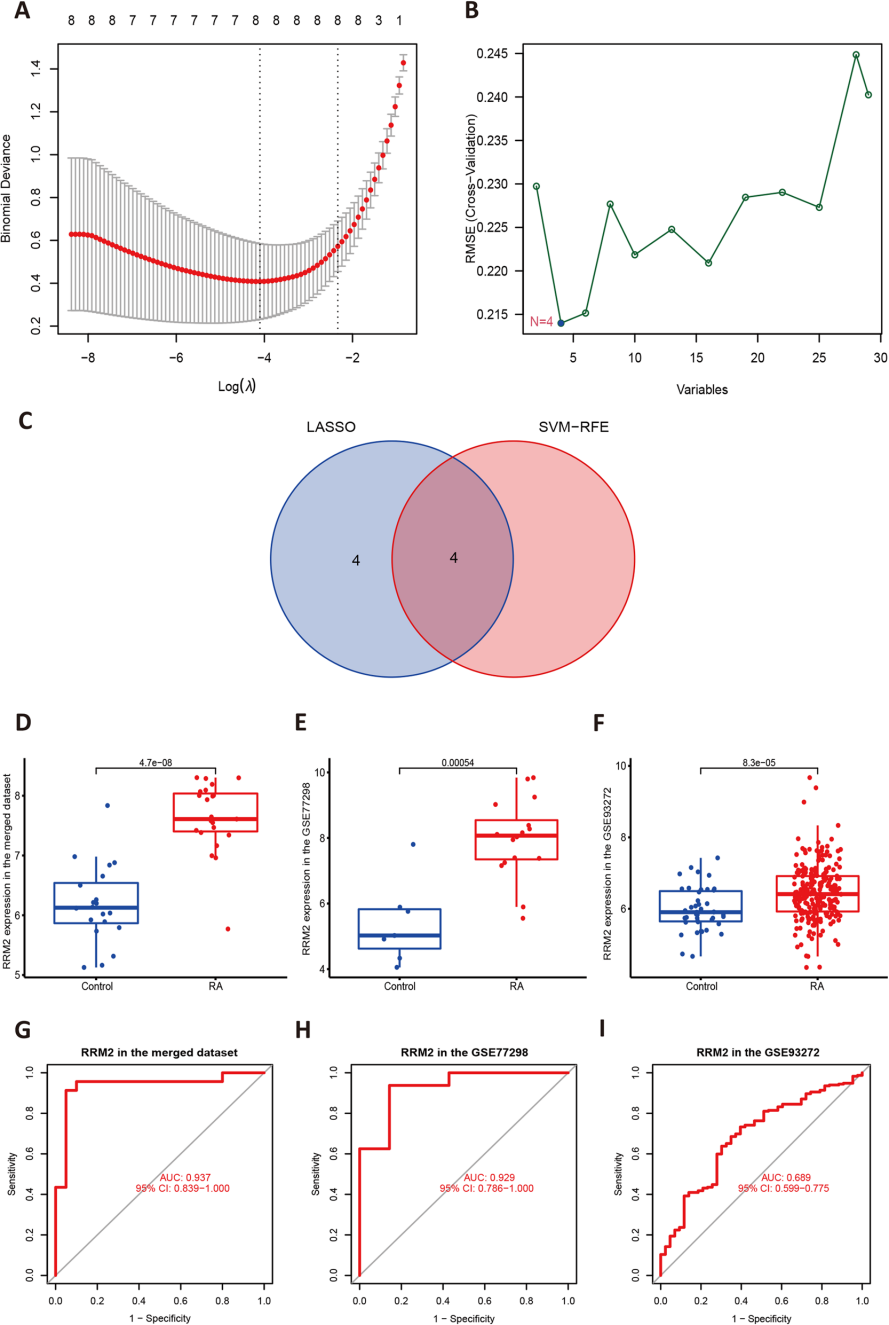
Screening of biomarkers and dataset validation. **A** 8 genes were filtered from the 29 overlapping genes by the LASSO logistic regression algorithm; **B** 4 genes were filtered from the 29 overlapping genes by the SVM-RFE algorithm; **C** 4 genes were intersected in the 2 algorithm; **D–F** the expression of RRM2 in different RA datasets: significantly increased (*P* < 0.05); **G**–**I** diagnostic efficacy of RRM2 in different RA datasets

With the algorithm of LASSO logistic regression, we totally collected 8 genes (GABARAPL1, EGFR, RRM2, DDR2, MAPK8, MEG3, GDF15, SCD) from the 29 DFRGs (Fig. 4A). Similarly, 4 duplicated genes (Fig. 4C), including GABARAPL1, RRM2, EGFR, and MAPK8, were identified by the SVM-RFE algorithm (Fig. 4B). The gene expression of GABARAPL1, RRM2, EGFR, and MAPK8 were subsequently verified in the GSE77298 and GSE93272 dataset (*P* < 0.05 was considered significant). The results showed that only RRM2 passed the validation (Fig. 4D–F). For the receiver operating characteristic (ROC) curve of RRM2, the area under the curve (AUC) in the merged GSE55235 and GSE55457 dataset was 0.937 (Fig. 4G); while the AUC of RRM2 in the GSE77298 and GSE93272 is 0.929 (Fig. 4H) and 0.689 (Fig. 4I), respectively. These implied that RRM2 had high diagnostic values in RA, which can be seem as the ferroptosis-related biomarker in RA.

### Immune infiltration analysis

**Fig. 5:**
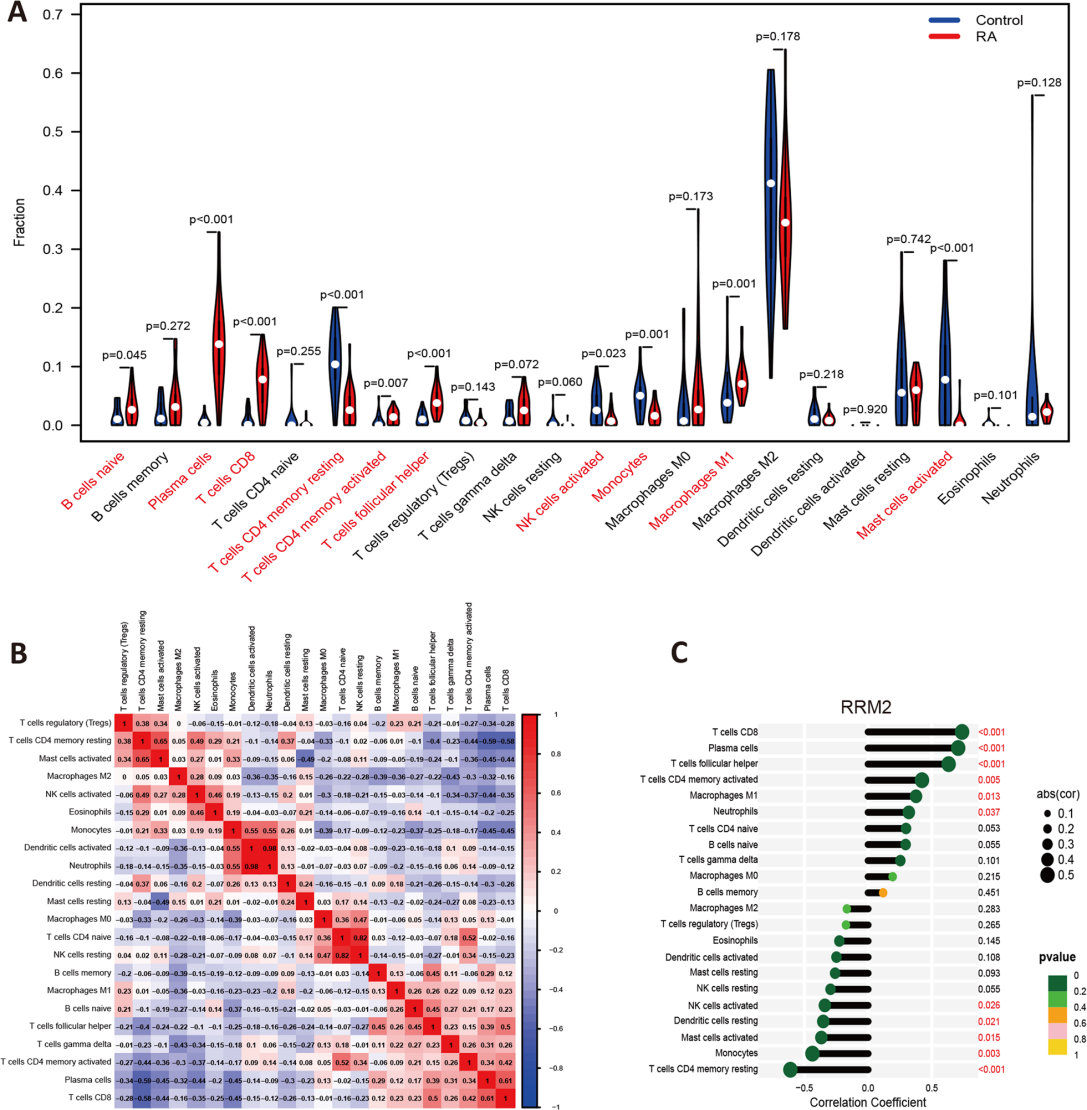
Analysis of immune cell infiltration in RA. **A** 10 types of immune cells (marked in red) were differentially distributed between groups in the violin diagram (*P* < 0.05); **B** The correlation heatmap of the 22 types immune cells (red: positive correlation; blue: negative correlation); C RRM2 was positively correlated with 6 type of infiltrating immune cells and negatively correlated with 5 type of cells in the lollipop chart (marked in red; *P* < 0.05)

In our study, 23 cases of synovial tissue from rheumatoid arthritis joint and 20 healthy controls (filtered with *P* < 0.05) were included for the immune infiltration analysis (22 types of immune cells) by the CIBERSORT algorithm. Totally, 10 types of cells were find differentially distributed between the groups (Fig. 5A, *P* < 0.05). They are B cells naive, Plasma cells, T cells CD8, T cells CD4 memory resting, T cells CD4 memory activated, T cells follicular helper, NK cells activated, Monocytes, Macrophages M1, and Mast cells activated. We also evaluate the potential correlations between all the 22 types of cells (Fig. 5B). In addition, the correlation analysis showed that RRM2 was positively correlated with the Neutrophils, Macrophages M1, T cells CD4 memory activated, T cells follicular helper, Plasma cells, and T cells CD8, while negatively related with NK cells activated, Dendritic cells resting, Mast cells activated, Monocytes, and T cells CD4 memory resting (Fig. 5C, *P* < 0.05).

### GSVA and GeneMANIA analysis of RRM2

**Fig. 6:**
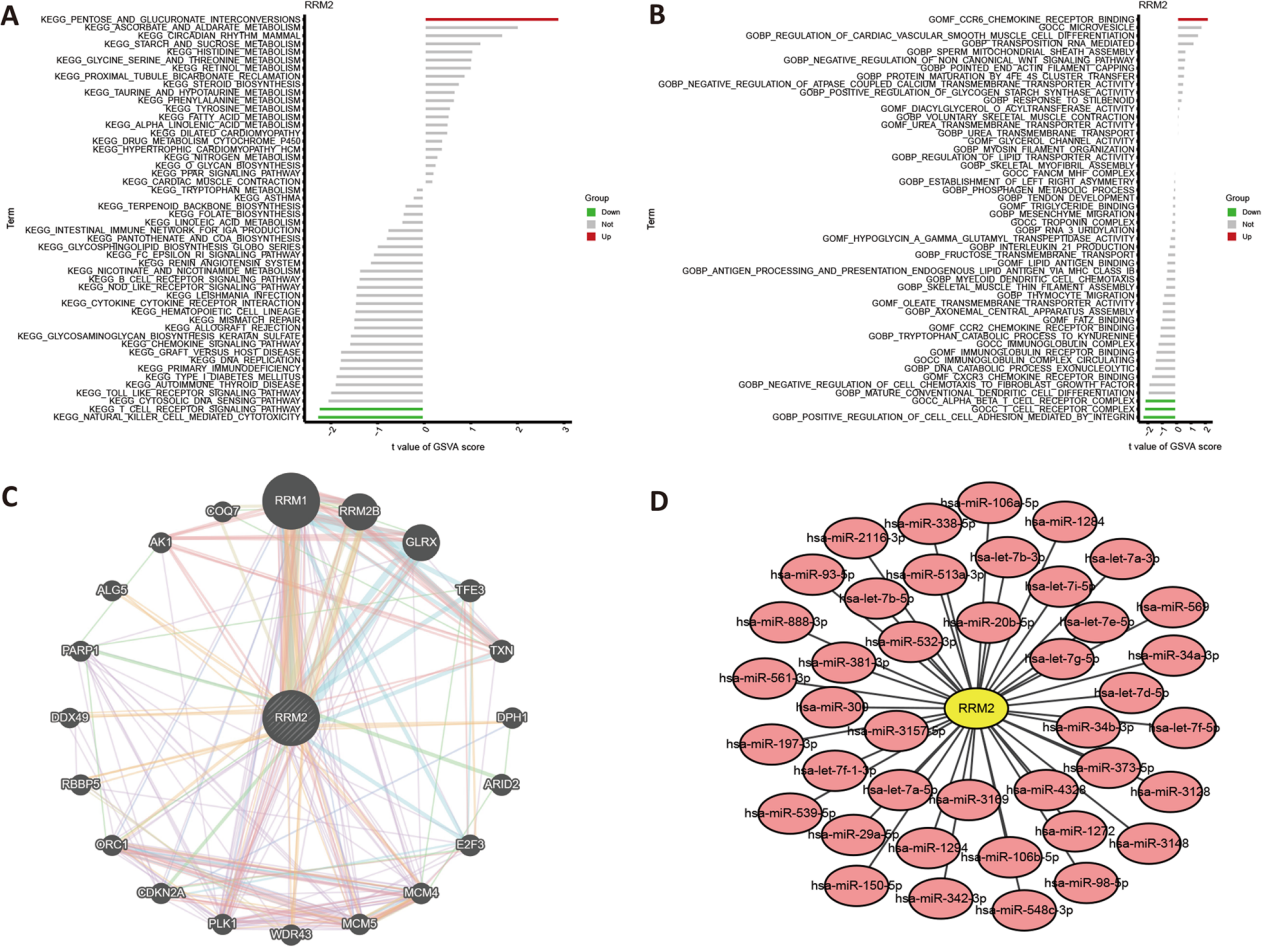
GSVA and gene interaction analysis of RRM2. **A** KEGG enrichments of RRM2 by GSVA; **B** GO enrichments of RRM2 by GSVA; **C** GeneMANIA analysis of RRM2; **D** RRM2-miRNAs network

Next, the molecular mechanism enriched in RRM2 were explored by GSVA. The KEGG results showed that the highly-expressed RRM2 was related to PENTOSE AND GLUCURONATE INTERCONVERSIONS, T CELL RECEPTOR SIGNALING PATHWAY, and NATURAL KILLER CELL MEDIATED CYTOTOXICITY (Fig. 6A). The results of MF and BP enrichments indicated that the highly expressed RRM2 was respectively related to the CCR6_CHEMOKINE_RECEPTOR_BINDING and the POSITIVE REGULATION OF CELL CELL ADHESION MEDIATED BY INTEGRIN; while the significant CC enrichments included the ALPHA BETA T CELL RECEPTOR COMPLEX and the T CELL RECEPTOR COMPLEX (Fig. 6B). The gene interaction involved in RRM2 is shown in Fig. 6C.

### Construction of RRM2-miRNAs network

MicroRNAs are significant gene regulators at the post transcriptional level. Based on information from online database (miRanda, miRDB, and TargetScan), we predicted miRNAs for RRM2. A total of 41 miRNAs were screened, and the gene-miRNA network was shown in Fig. 6D.

### Cell viability assay

**Fig. 7:**
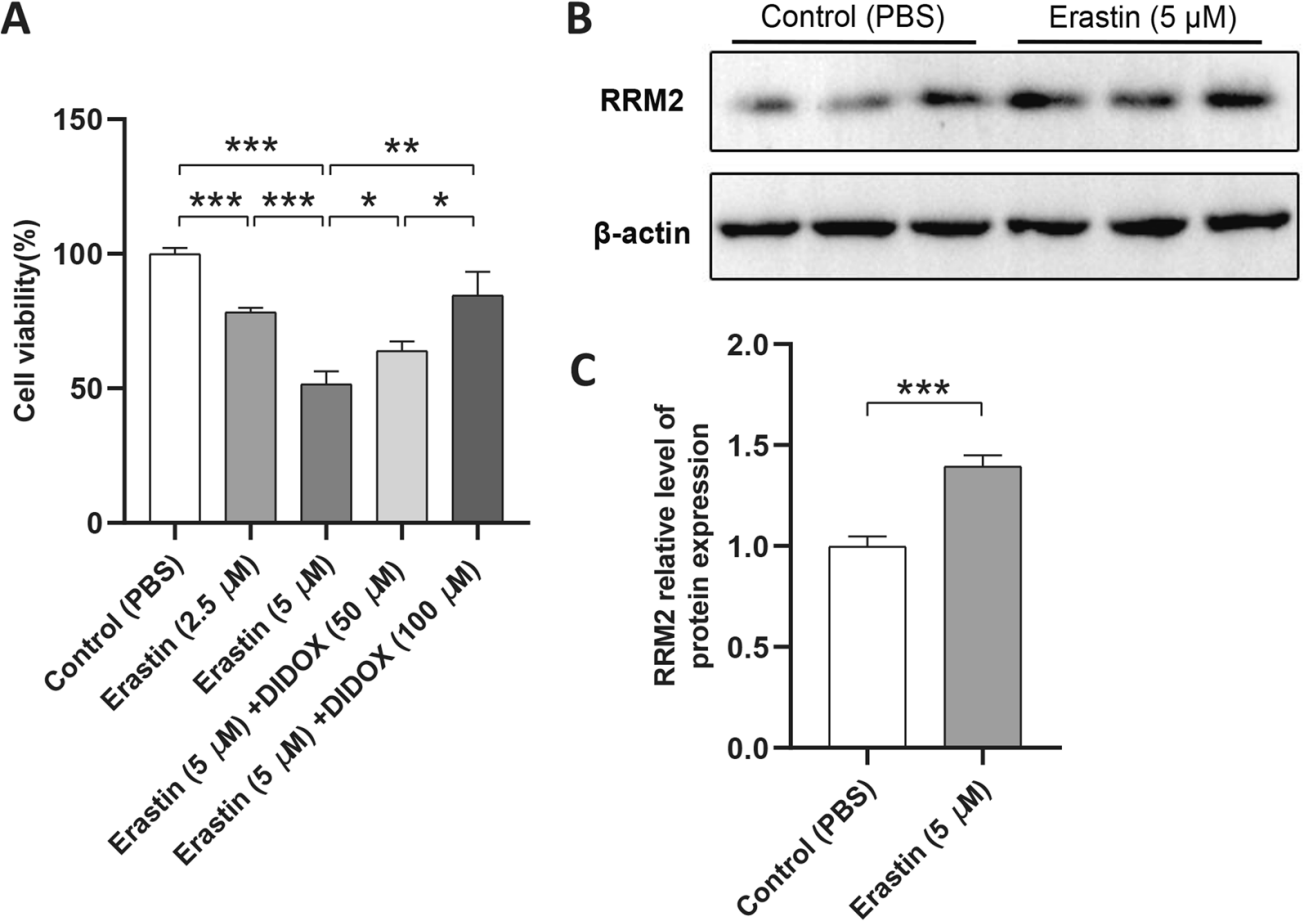
Construction and validation of ferroptosis model in vitro **A** The viability of C28/I2 cells by CCK8 assay; **B** Western blot validation of RRM2 in vitro (β-actin was used as the internal control); **C** the expression levels of RRM2 under Erastin treatment were significantly increased when compared with control. *P < 0.05, **P < 0.01, ***P < 0.001

Using the Erastin (ferroptosis inducer), an in vitro model in human chondrocytes (C28/I2 cells) was established in our study. The results suggested that the Erastin (5 μM) significantly reduced the viability of C28/I2 cells (*P* < 0.05, Fig. 7A). In a concentration-dependent manner, DIDOX (inhibitor of RRM2) [36] restored the decreased viability of Erastin-treated C28/I2 cells (*P* < 0.05, Fig. 7A), implying the involvement of elevated RRM2 in ferroptosis.

### Western blot

Western blot was selected for further validation of RRM2 after the establishment of ferroptosis model in vitro. Comparing to the controls, the expression of RRM2 was significantly upregulated under the Erastin treatment group (*P* < 0.05, Fig. 7B and C; Additional files 7 and 8). This result suggests that the abnormal accumulation of RRM2 is indeed associated with the increased ferroptosis in RA.

## Discussion

Rheumatoid arthritis is a chronic autoimmune disease characterized by destructive and symmetrical joint lesions, joint synovitis, and is dominated by symptoms such as joint deformities, morning stiffness, and temporomandibular arthritis of the hand, foot, wrist, and ankle [37], which not only decrease the motor function of patients, but damage respiratory, renal, and cardiac systems [38], so that the life and work of patients are severely affected. If the patient's condition is not controlled promptly and effectively, it will trigger complications such as Sjogren's syndrome, pericarditis, anemia, and necrotizing vasculitis [39], which threaten their life safety. Currently, the pathogenesis of RA in the medical field is still under investigation, and there is an increasing number of scholars who believe that ferroptosis is the main factor triggering rheumatoid arthritis.

In this study, gene expression profiling of synovial tissues (healthy controls and RA paitients) were downloaded from the NCBI-GEO database. After data processing, the DFRGs were screened out. Based on them. GO, KEGG, and DO enrichments were performed. Besides, the machine learning algorithms were applied to further screen the ferroptosis related markers in RA. Through dataset and experimental validation, biomarkers was finally determined, the GSVA analysis of which was conducted and the regulatory miRNAs were predicted. In addition, the infiltrated immune cells between RA and controls were evaluated, and the correlation between them was also calculated.

As a result, we totally identified 29 differentially expressed genes related to ferroptosis (DFRGs) in the merged datasets (GSE55235 and GSE55457) by integrated bioinformatics analysis. With the clusterProfiler package in R, we found that these genes were significantly enriched in the inherited metabolic disorder and the FoxO signaling pathway. The machine learning algorithms (LASSO and SVM-RFE) calculated 4 hub genes from them, which are respectively GABARAPL1, RRM2, EGFR, and MAPK8. To find markers with higher diagnostic value in clinical practice, we performed validation in both synovial tissues (GSE77298) and blood (GSE93272) derived datasets. Ultimately, only RRM2 (*P* < 0.05) passed the additional verifications. According to our analysis, RRM2 was significantly up-regulated in synovial tissue and peripheral blood of RA suffers, having a good performance as a the diagnostic marker (the AUC was 0.937 and 95% CI: 0.839–1.000 in the merged GSE55235 and GSE55457; the AUC was 0.929 and 95% CI: 0.786–1.000 in the GSE77298; the AUC was 0.689 and 95% CI: 0.599–0.775 in the GSE93272). In our in vitro model (C28/I2 cells) constructed with a ferroptosis inducer (Erastin), the expression of RRM2 protein was significantly elevated in the intervention group measured by western blot, and the inhibition of which by DIDOX treatmet can markedly reverse the decrease of cell viability caused by the Erastin induced ferroptosis. Thus, we boldly conclude that RRM2 is abnormally highly expressed in rheumatoid arthritis samples and its pharmacological blockade can attenuate chondrocyte ferroptosis in vitro. Because of its biological function and diagnostic efficacy, RRM2 may serve as a crucial target for ferroptosis related mechanisms in rheumatoid arthritis.

Ribonucleotide reductase (RR), the only rate limiting enzyme of intracellular DNA synthesis, has an important role in the process of nucleic acid metabolism. RR catalyzes the reduction of nucleoside diphosphates to deoxyribonucleoside diphosphates, a rate limiting step in the biosynthesis of deoxyribonucleoside triphosphates [40]. The RR includes two subunits, M1 and M2, both of which are in a dimeric structure. RRM1 has a site that binds to substrates and allosteric effectors, and contains sulfhydryl groups that directly donate electrons. It controling the specificity of substrates and the switch of enzymatic activity, is a tumor suppressor gene. RRM2, an iron sulfur protein that participates in catalytic reactions by forming specific radicals through the phenyl ring of tyrosine residues, is a major regulatory element of RR activity [41]. RRM2 is involved in the biosynthesis of deoxyribonucleoside triphosphate (dNTP) and is an indispensable key enzyme in the process of DNA synthesis and repair. Studies have found that RRM2 is overexpressed in a variety of malignant tumors, identifing as a marker for the prediction and prognosis [42]. But so far, research on its use in osteoarticular diseases has remained in its infancy. Our results suggest that RRM2 has important value in the diagnosis and treatment of rheumatoid arthritis, expecting it to play a role in the new research breakthrough.

With the establishment of disease models and the development molecular biology technology, it has been proved that infection, environmental factors, immune abnormalities, etc. are closely related to the pathogenesis of rheumatoid arthritis [43, 44]. Among them, immune factors have been studied more in recent years, and it has been found that A variety of immune cells regulate each other and form a complex network to participate in the occurrence and development of RA [45]. In our research results, we found that 10 types of immune cells were distributed differently between groups, four of which were T lymphocytes (*P* < 0.05). They are specifically T cells CD8, T cells CD4 memory resting, T cells CD4 memory activated, and T cells follicular helper. In general, T lymphocytes are derived from lymphoid stem cells in the bone marrow, develop and mature within the thymus, and primarily mediate adaptive cellular immune responses. According to the expression of CD4 and CD8 molecules, T cells can be divided into CD4 + T cells and CD8 + T cells, wherein CD4 + T cells include Th1, Th2, Th17 and Treg cells [46]. Based on the existing studies, a significant imbalance between Th1 and Th2 cells was detected in RA patients and animal models, and Th17 in the active RA patients was significantly higher than that in the inactive and healthy controls [47]. Currently, targeted therapeutics (e.g., abatacept, etanercept, tofacitinib, etc.) [48–50] targeting immune cells or the cells and inflammatory factors produced by them have been applied in the clinic and have achieved better outcomes; But the involvement of immune cells and their secreted cytokines in the mechanisms and interactions with RA pathogenesis remain incompletely understood, and in-depth studies will provide novel strategies for early detection, disease assessment and treatment of RA.

Regrettably, the present analysis suffers from the inadequacy of a relatively small sample size, which may limit our judgment to some extent. Therefore, these potential mechanisms still need to be further verified.

## Conclusion

Datasets of rheumatoid arthritis and ferroptosis related genes were downloaded from the public database. Through a comprehensive bioinformatics analysis, we have preliminarily explored the RA pathological mechanism related to ferroptosis. Enrichment analysis of the differentially expressed genes involving ferroptosis in RA suggested a closed association with FoxO signaling pathway and inherited metabolic disorder. Among them, RRM2 can be seem as the ferroptosis-related biomarker. RRM2 is not only differentially expressed in the dataset of patients’ synovium tissues and blood samples, which has good disease diagnostic value, but significantly improves the damaged cell viability after inhibiting its function in the ferroptosis model in vitro. Its modulators, such as the potential miRNAs and related immune cells, have also been analyzed in this study.

## Supplementary Information

**Additional file 1:** Driver in ferroptosis-related genes.

**Additional file 2:** Suppressor in ferroptosis-related genes.

**Additional file 3:** Marker in ferroptosis-related genes.

**Additional file 4:** Details of the 635 DEGs in RA (255 down-regulated; 380 up-regulated).

**Additional file 5:** Details for differentially expressed ferroptosis-related genes.

**Additional file 6:** GO enrichment results for the 29 DEGs.

**Additional file 7:** Original, unprocessed versions for WB: RRM2.

**Additional file 8:** Original, unprocessed versions for WB: β-actin.

## Data Availability

The original contributions presented in the study are included in the article/Additional files, further inquiries can be directed to the corresponding authors.

## Abbreviations

RA: Rheumatoid arthritis
GEO: Gene expression omnibus
DEGs: Differentially expressed genes
GO: Gene ontology
MF: Molecular function
BP: Biological process
CC: Cellular component
KEGG: Kyoto encyclopedia of genes and genomes
FRGs: Ferroptosis-related genes
DFRGs: Differentially expressed genes related to ferroptosis
GSEA: Gene set enrichment analysis
GSVA: Gene set variation analysis
LASSO: Least absolute shrinkage and selection operator
SVM-RFE: Support vector machine-recursive feature elimination
GPX: Glutathione peroxidase
GSH: Glutathione
ROS: Reactive oxygen species
ROC: Receiver operating characteristic
