# Supplementary material for: Identification of ferroptosis related markers by integrated bioinformatics analysis and In vitro model experiments in rheumatoid arthritis
Source: BMC Med Genomics. 2023 Jan 30;16:18. doi: 10.1186/s12920-023-01445-7 (PMC9887825; doi:10.1186/s12920-023-01445-7)
Supplement: Supplementary file 5 — Additional file 5: Details for differentially expressed ferroptosis-related genes. [file 12920_2023_1445_MOESM5_ESM.docx]

**Supplementary file 5** Details for differentially expressed ferroptosis-related genes

| Type | Genes | Count |
| --- | --- | --- |
| Driver | GABARAPL1; EGFR; EGR1; DDR2; MAPK8; MEG3; ALOX5; ATF3; IDO1; PPARG; DUOX2; IL6 | 12 |
| Suppressor | PTGS2 | 1 |
| Marker | RRM2; ZFP36; CDKN1A; JUN; IDH2; NR4A1; GDF15; KIF20A; FABP4; ARNTL; CP; SCD; ADIPOQ; PDK4; EZH2; RARRES2 | 16 |
