# Supplementary material for: Identification of ferroptosis related markers by integrated bioinformatics analysis and In vitro model experiments in rheumatoid arthritis
Source: BMC Med Genomics. 2023 Jan 30;16:18. doi: 10.1186/s12920-023-01445-7 (PMC9887825; doi:10.1186/s12920-023-01445-7)
Supplement: Supplementary file 6 — Additional file 6: GO enrichment results for the 29 DEGs. [file 12920_2023_1445_MOESM6_ESM.docx]

**Supplementary file 6** GO enrichment results for the 29 DEGs

| **Category** | **ID** | **Term** | **Genes** | **Adj-pval** |
| --- | --- | --- | --- | --- |
| BP | GO:0045444 | fat cell differentiation | ZFP36/ALOX5/NR4A1/PTGS2/PPARG/FABP4/ARNTL/ADIPOQ/RARRES2/IL6 | 1.70338E-09 |
| BP | GO:0031667 | response to nutrient levels | GABARAPL1/EGFR/ZFP36/CDKN1A/MAPK8/JUN/GDF15/ATF3/PTGS2/PPARG/ADIPOQ/PDK4 | 1.70338E-09 |
| BP | GO:0045598 | regulation of fat cell differentiation | ZFP36/ALOX5/PTGS2/PPARG/ARNTL/ADIPOQ/RARRES2/IL6 | 2.0124E-08 |
| BP | GO:0042594 | response to starvation | GABARAPL1/ZFP36/CDKN1A/MAPK8/JUN/ATF3/PPARG/PDK4 | 2.50253E-07 |
| BP | GO:0006979 | response to oxidative stress | EGFR/MAPK8/JUN/ALOX5/PTGS2/ARNTL/DUOX2/ADIPOQ/EZH2/IL6 | 2.50253E-07 |
| BP | GO:0071496 | cellular response to external stimulus | GABARAPL1/EGFR/CDKN1A/MAPK8/JUN/ATF3/PTGS2/PPARG/PDK4 | 2.50253E-07 |
| BP | GO:0007623 | circadian rhythm | EGFR/EGR1/MAPK8/JUN/PPARG/ARNTL/ADIPOQ/EZH2 | 2.50253E-07 |
| BP | GO:0062197 | cellular response to chemical stress | EGFR/MAPK8/JUN/ALOX5/PTGS2/PPARG/ARNTL/EZH2/IL6 | 4.88299E-07 |
| BP | GO:0048660 | regulation of smooth muscle cell proliferation | EGFR/CDKN1A/JUN/PTGS2/PPARG/ADIPOQ/IL6 | 1.23848E-06 |
| BP | GO:0048659 | smooth muscle cell proliferation | EGFR/CDKN1A/JUN/PTGS2/PPARG/ADIPOQ/IL6 | 1.23848E-06 |
| CC | GO:0031965 | nuclear membrane | EGFR/ALOX5/NR4A1/PTGS2 | 0.088312929 |
| CC | GO:0005667 | transcription regulator complex | JUN/NR4A1/PPARG/ARNTL | 0.129460816 |
| CC | GO:0005635 | nuclear envelope | EGFR/ALOX5/NR4A1/PTGS2 | 0.129460816 |
| CC | GO:0005788 | endoplasmic reticulum lumen | PTGS2/CP/IL6 | 0.129460816 |
| CC | GO:1990204 | oxidoreductase complex | RRM2/DUOX2 | 0.129460816 |
| CC | GO:0031983 | vesicle lumen | EGFR/ALOX5/RARRES2 | 0.129460816 |
| CC | GO:0005641 | nuclear envelope lumen | ALOX5 | 0.129460816 |
| CC | GO:0016324 | apical plasma membrane | EGFR/DDR2/DUOX2 | 0.129460816 |
| CC | GO:0005845 | mRNA cap binding complex | ZFP36 | 0.129460816 |
| CC | GO:0043020 | NADPH oxidase complex | DUOX2 | 0.129460816 |
| MF | GO:0016702 | oxidoreductase activity, acting on single donors with incorporation of molecular oxygen, incorporation of two atoms of oxygen | ALOX5/IDO1/PTGS2 | 0.000601778 |
| MF | GO:0016701 | oxidoreductase activity, acting on single donors with incorporation of molecular oxygen | ALOX5/IDO1/PTGS2 | 0.000601778 |
| MF | GO:0061629 | RNA polymerase II-specific DNA-binding transcription factor binding | GABARAPL1/JUN/NR4A1/PPARG/ARNTL | 0.00233007 |
| MF | GO:0140297 | DNA-binding transcription factor binding | GABARAPL1/JUN/NR4A1/PPARG/ARNTL | 0.006009326 |
| MF | GO:0036041 | long-chain fatty acid binding | PPARG/FABP4 | 0.006938269 |
| MF | GO:0051213 | dioxygenase activity | ALOX5/IDO1/PTGS2 | 0.00877089 |
| MF | GO:0031625 | ubiquitin protein ligase binding | GABARAPL1/EGFR/CDKN1A/JUN | 0.019945154 |
| MF | GO:0020037 | heme binding | IDO1/PTGS2/DUOX2 | 0.019945154 |
| MF | GO:0044389 | ubiquitin-like protein ligase binding | GABARAPL1/EGFR/CDKN1A/JUN | 0.019945154 |
| MF | GO:0046906 | tetrapyrrole binding | IDO1/PTGS2/DUOX2 | 0.019945154 |
