# Supplementary figures and images for: Identification of ferroptosis related markers by integrated bioinformatics analysis and In vitro model experiments in rheumatoid arthritis
Source: BMC Med Genomics. 2023 Jan 30;16:18. doi: 10.1186/s12920-023-01445-7 (PMC9887825; doi:10.1186/s12920-023-01445-7)

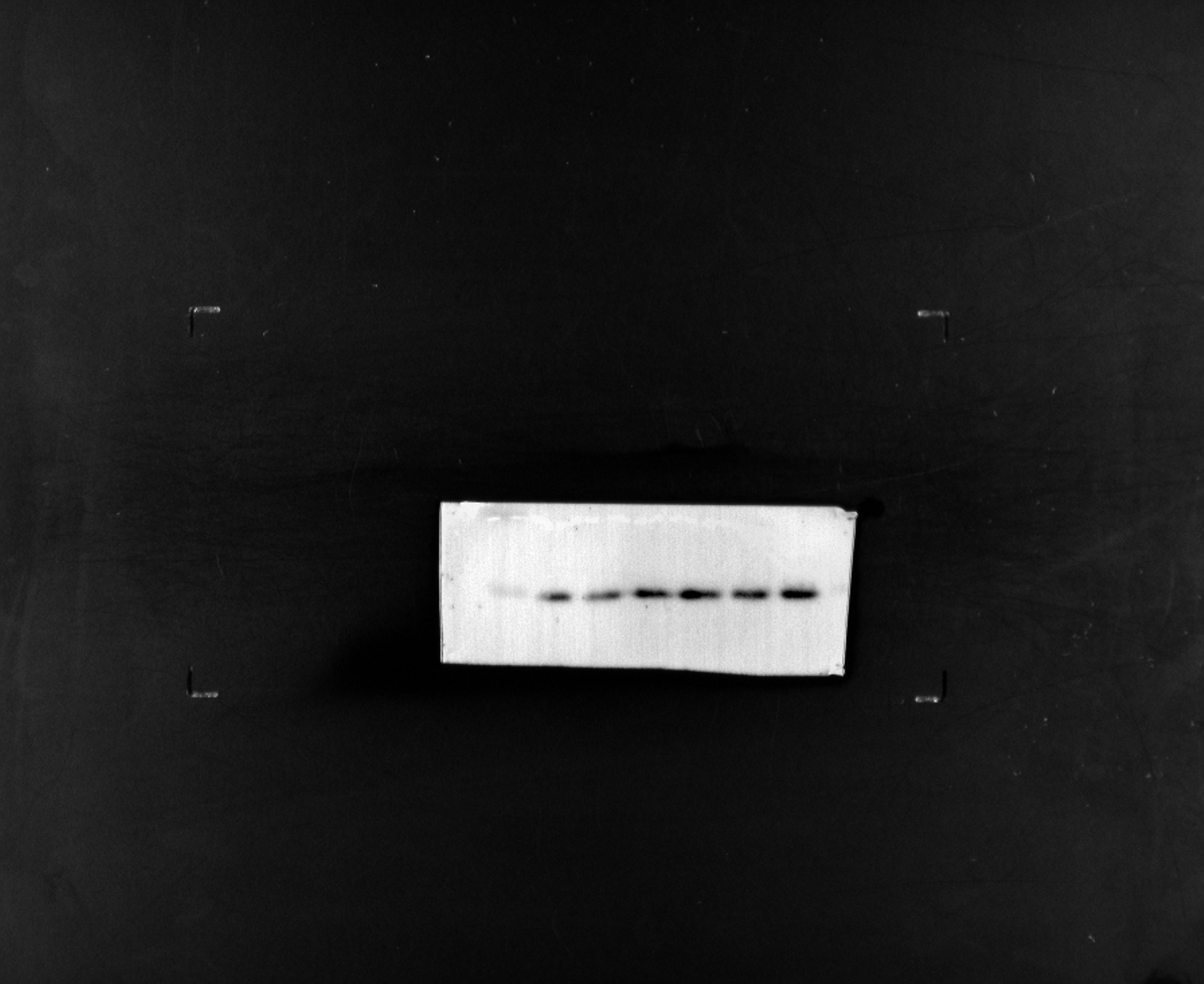

Supplement: Supplementary file 7 — Additional file 7: Original, unprocessed versions for WB: RRM2. [file 12920_2023_1445_MOESM7_ESM.tif]

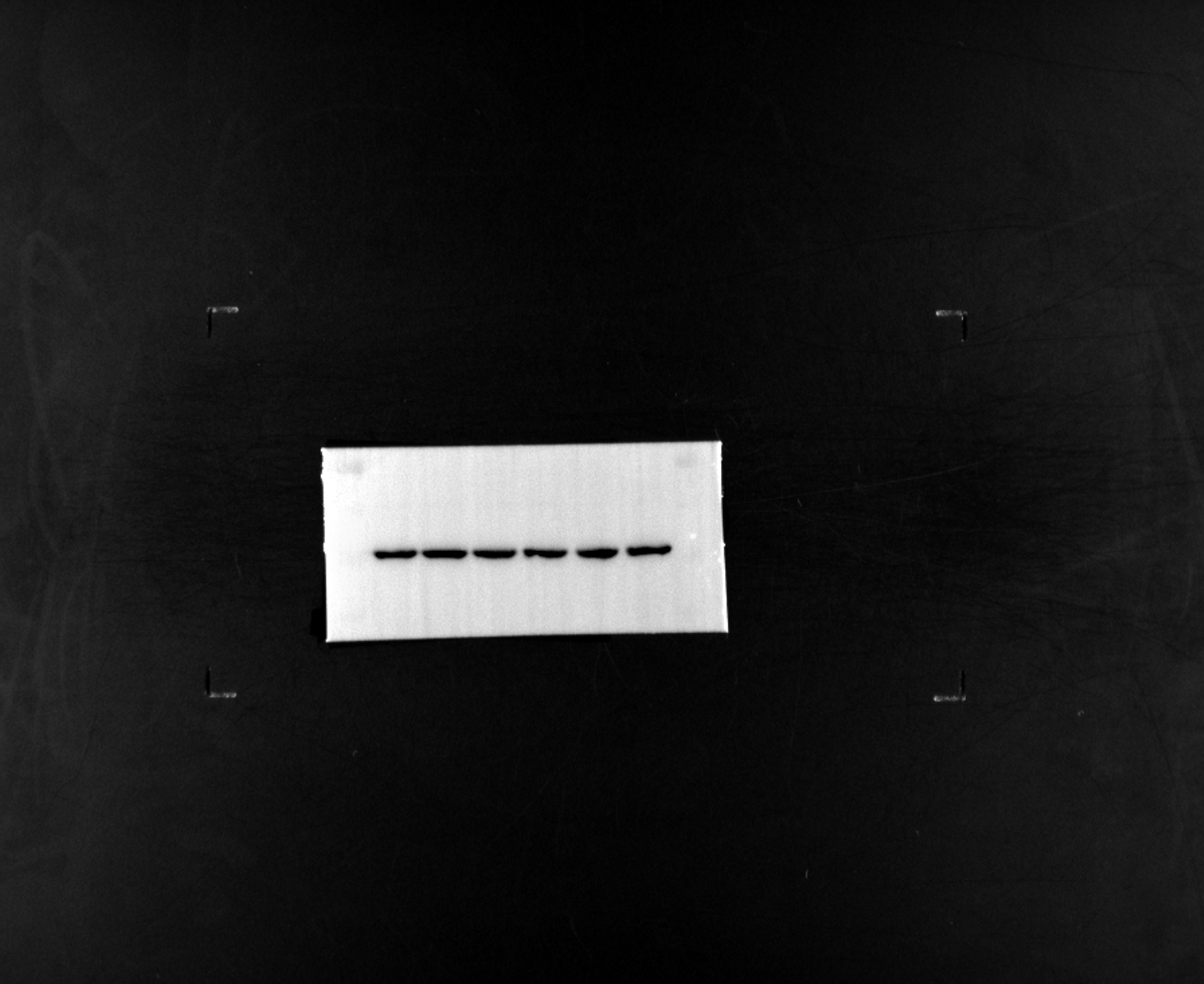

Supplement: Supplementary file 8 — Additional file 8: Original, unprocessed versions for WB: β-actin. [file 12920_2023_1445_MOESM8_ESM.tif]
